# Case report: Crigler-Najjar syndrome type 1 in Croatia—more than a one in a million: a case series

**DOI:** 10.3389/fped.2023.1276349

**Published:** 2023-10-19

**Authors:** Matea Kovačić Perica, Ivana Todorić, Nedo Marčinković, Petra Džepina, Mirna Natalija Aničić, Anna Mrzljak, Jurica Vuković

**Affiliations:** ^1^ Department of Pediatrics, University Hospital Center Zagreb, Zagreb, Croatia; ^2^ School of Medicine, University of Zagreb, Zagreb, Croatia; ^3^ Department of Gastroenterology and Hepatology, University Hospital Center Zagreb, Zagreb, Croatia

**Keywords:** crigler-Najjar syndrome, bilirubin UDP-glucuronosyltransferase, UGT1A1, hyperbilirubinemia, Croatia, case series, case report

## Abstract

Crigler-Najjar syndrome (CNS) is an exceedingly rare autosomal recessive disease with an estimated incidence of 1 in a million live births. CNS type 1 (CNS1) is the most severe form, characterized by severe unconjugated hyperbilirubinemia since birth due to the absence of hepatic uridine 5'-diphosphate glucuronyltransferase (UGT1A1) activity. Daily phototherapy (PT) and liver transplant (LT) are the mainstays of therapy. Here, we present a higher-than-expected incidence of CNS1 in Croatia (6,1 in a million). In the last 31 years, we treated eight CNS1 patients from five families with no reported consanguinity. Four patients are descendants of an isolated enclave in Kosovo with a small gene pool and a high potential for inbreeding. Severe unconjugated hyperbilirubinemia was verified in a neonatal period and PT was initiated. Four patients underwent LT from living-related donors. One of them had unsuccessful hepatocyte transplantation earlier. LT was successful in three patients, and one patient died due to primary graft dysfunction. Four patients are currently treated with 9–12 h daily PT with inconsistent disease control, and gradually increasing bilirubin. One patient developed kernicterus before LT, while others have normal psychomotor development and no neurologic impairment. Genetic testing of the UGT1A1 gene in six patients from three families revealed three different homozygous mutations (c.722_723 delAG, c.717_718 delAG, and c.1021 C >T), all previously described in other populations. There is a possibility of the founder effect as an explanation for the higher incidence of CNS1 in at least a subgroup of Croatians.

## 1. Introduction

Crigler-Najjar syndrome (CNS) is an extremely rare autosomal recessive disorder of bilirubin conjugation (1) caused by the deficiency of hepatic uridine 5'-diphosphate glucuronyltransferase (UGT1A1) (2). Glucuronidation is a requisite for bilirubin excretion, and the absence of functional UGT1A1 leads to the accumulation of unconjugated bilirubin (UCB) in serum (2, 3).

Residual UGT1A1 activity and its inducibility by phenobarbital (PB) exposure allow classification into clinical variants: the most severe type 1 (CNS1; absence of UGT1A1 activity), type 2 (CNS2; 4 to 10% residual UGT1A1 activity), and Gilbert syndrome (GS; 20 to 30% residual UGT1A1 activity) (4).

In patients with CNS1, severe and persistent unconjugated hyperbilirubinemia begins in the neonatal period and continues throughout life. These patients are at high risk for developing bilirubin-induced neurologic dysfunction (BIND), ranging from mild and reversible neurologic manifestations to irreversible encephalopathy, commonly known as kernicterus (5). They do not respond to PB therapy and require 7 or more hours of high-intensity phototherapy (PT) daily to maintain serum bilirubin within safe margins until liver transplant (LT), the current curative method (4, 6–8).

CNS is an ultra-rare disease with an estimated incidence of 1 in 1 000 000 newborns in Western countries (9). The incidence is higher in countries with a high consanguinity rate or a significant founder effect (4). Consequently, in Croatia, a country with just below 4 million inhabitants, roughly 35 000 live births per year, and a low consanguinity rate, we would expect CNS patients to be extremely rare, with the newly diagnosed patient once in 25 to 30 years. Surprisingly, we have treated 8 CNS1 patients in the last 31 years. In that period (1992–2022), there were 1 314 533 live births in Croatia (The Croatian Bureau of Statistics data), so the incidence of CNS1 in Croatia is 6,1 in 1 000 000 live births.

Here, we present 8 patients with CNS1 who were at some point treated in our tertiary care pediatric hepatology center at the University Hospital Center Zagreb in the last 31 years (born between 1992 and 2022). To our knowledge, we included all patients diagnosed with CNS1 in Croatia during that period.

The Ethical Committee of the University Hospital Center Zagreb approved the study, and patients or their parents provided an informed consent.

## 2. Case presentation

Patients developed progressive jaundice in the first days of life, which persisted and worsened in the following weeks. Drowsiness and feeding difficulties were present. Stools and urine were of normal color. All patients were born at term from uncomplicated pregnancies with normal birth weight and had no prior difficulties.

### 2.1. Personal and family history

Our patients were born in 5 families, with no reported consanguinity. The older brother of patient 2 was born in Kosovo, treated elsewhere, and died at the age of 6 months because of severe hyperbilirubinemia without an established diagnosis at the time of death. Patient 3 is a 5-year older sister of a male patient 4 (family A). Patients 6 (female), 7 (male), and 8 (female) (family B) are siblings and only children of a mother with confirmed GS (genotype TA7/TA7). Other patients are unrelated, and their family histories are unremarkable.

### 2.2. Physical examination and laboratory tests upon admission

Patients 6, 7, and 8 were under our surveillance since the first weeks of life, whereas others were initially treated in other centers and referred to ours later because of unremitting jaundice. Apart from somnolence, their neurologic exams were normal. No dysmorphic features, hepatomegaly, or splenomegaly were noted. There were no signs of bleeding diathesis.

Persistently high total bilirubin (TB) and UCB serum levels with no signs of hemolysis were verified in all patients. On admission and before PT treatment, maximal UCB values ranged from 374 to 521 umol/L. They had normal liver enzymes, albumin concentration, and coagulation workup.

Genetic testing for GS was performed as the next step in five patients. Polymerase chain reaction (PCR) amplification of the TATA-box element in the promoter of *UGT1A1* revealed UGT1A1*28 (TA7/TA7) genotype consistent with GS in three patients and normal (TA6/TA6) genotype in the remaining two.

The available findings could not explain the persistent UCB requiring daily PT. Direct sequencing of the entire coding sequence and splice sites of the UGT1A1 was performed in seven patients and revealed homozygous mutations consistent with CNS1 in all (Table 1). Unfortunately, the results of patient 1 are lost. Genetic testing, as it was not widely available at the time, was not performed in patient 2. The chromatographic analysis of bile confirmed her diagnosis, which revealed the absence of conjugated bilirubin.

**Table 1:** Patient characteristics.

Patient No	Relationship	Sex	Mutation	Mutant protein	Exon	Mutation type	TA polymorphism
1	Unrelated	M	Result lost	N/A	N/A	N/A	N/A
2	Unrelated	F	Not performed	N/A	N/A	N/A	N/A
3	Family A	F	c.717_718delAG	p.Q239fsX256	1	Frameshift	TA7/TA7
4	Family A	M	c.717_718delAG	p.Q239fsX256	1	Frameshift	TA7/TA7
5	Unrelated	M	c.722_723delAG	p.Glu241Glyfs*16	1	Frameshift	TA7/TA7
6	Family B	F	c.1021C >T	p.(Arg341*)	3	Nonsense	TA6/TA6
7	Family B	M	c.1021C >T	p.(Arg341*)	3	Nonsense	TA6/TA6
8	Family B	F	c.1021C >T	p.(Arg341*)	3	Nonsense	N/A

* Indicates a translation stop codon.

### 2.3. Treatment

PT was initiated in all patients following the verified pathologic hyperbilirubinemia in the first weeks of life and continued at home (except in patient 1). Patient 2 underwent an exchange transfusion 2 times prior to PT. In infancy, 6 to 12 h of PT daily was sufficient to keep the UCB levels below neurotoxic levels (< 340 umol/L). A phenobarbital test was performed in all patients, with no significant effect on TB levels.

Patient 1 was initially treated in two other centers. During the newborn period, he was treated with intermittent PT and PB. He was then transferred to an international center, diagnosed with CNS, and treated with daily PT. Unfortunately, at the age of 9 months, following his return to Croatia, PT was stopped, and he did not receive any therapy for years. During that period, his parents noticed he was extremely icteric and agitated. At the age of 5 years, there was an abrupt developmental and speech regression with postural instability and choreoathetosis. Upon referral to our center, at the age of 5 years, he was diagnosed with CNS1, and daily PT was initiated.

All patients had transient exacerbations of hyperbilirubinemia, usually due to intercurrent respiratory or febrile illnesses, fasting, or nonadherence to PT. If needed, they were treated with intensified PT, parenteral rehydration, and albumin infusions. Despite relatively uniform PT conditions, UCB levels gradually increased with advancing age and reached potentially neurotoxic levels, which prompted us to perform LT from living-related donors in 4 patients. The last laboratory results before LT are listed in Table 2.

**Table 2:** Laboratory results of our patients.

Patient	Total bilirubin (umol/L)	Direct bilirubin (umol/L)	AST (U/L)	ALT (U/L)	GGT (U/L)	ALP (U/L)	Albumin (g/L)	Hct (%)	WBC (x10^9^/L)	Plt (x10^9^/L)
1	486	N/A	N/A	N/A	N/A	N/A	N/A	N/A	N/A	N/A
2	373	37	45	45	13	293	N/A	31,1	6,6	290
3	393	6	57	94	16	228	38,9	36,4	9,3	294
4	385	N/A	66	57	24	249	41	N/A	6,8	189
5	234	23	47	73	26	285	44,1	36,3	11,1	284
6	456	10	72	96	26	229	N/A	35,6	9,9	244
7	370	N/A	52	89	25	179	N/A	35,2	10,5	246
8	290	N/A	77	143	36	238	42	36,1	11,9	774

For patients 1–4 last values before liver transplant are listed, and for non-transplanted patients 5–8 values from the last follow-up.

Patient 1 had extreme hyperbilirubinemia at the age of 9.5 years (TB of 486 umol/L) and did not tolerate PT. He underwent hepatocyte transplantation (HT). The procedure was unsuccessful, and without PT, TB levels soon reached the ones before the procedure. Liver biopsy results were unremarkable. Subsequently, 6 months later, he underwent orthotopic segmental LT (left lobe). Similarly, patient 2 had a gradual increase in TB which reached 600 umol/L, and at the age of 5.5 years, the orthotopic segmental LT (left lobe) was performed. Patients 3 and 4 at the age of 9 and 7 years underwent auxiliary partial orthotopic liver transplantation (APOLT) using the left lobe of the living donor.

### 2.4. Outcome and follow-up

LT succeeded in 3 patients (1, 3, and 4). The immunosuppression protocol was based on steroids and tacrolimus in 2 patients, with the addition of mycophenolate mofetil in patient 1 because of suspected early chronic graft rejection. After LT, PT was discontinued, levels of TB and liver enzymes soon normalized and remained stable. Now, 21, 13, and 11 years after LT, they are on a low-dose tacrolimus immunosuppressive therapy with normal liver tests.

In patient 2, the LT procedure was successful, but unfortunately, she developed primary graft non-function resulting in her death 4 days later.

**Figure 1:**
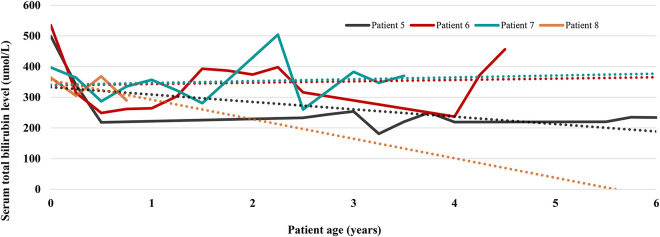
Serum total bilirubin trends in 4 patients currently treated with phototherapy. Each patient is represented by one line chart, and dotted lines in corresponding colors are trendlines showing the prevailing direction of serum total bilirubin levels.

Four patients (patients 5, 6, 7, and 8, currently at the age of 6, 4.5, 3.5 years, and 9 months) are currently managed by PT. Figure 1 depicts the trends of TB values in these patients. While the ascending trendlines in 2 older patients come as no surprise and reflect poor compliance to PT, reassuring is the descending trendline in the oldest patient with strict adherence to PT. The second descending trendline in the youngest patient is expected because infants generally require shorter PT and tolerate it better. Laboratory results from the last follow-up are listed in Table 2. In patient 5, very good disease control is achieved with PT through the night (9–11 h) using homemade blue light-emitting diode (LED) lamps. His TB levels are between 180 and 230 umol/L, alanine aminotransferase (ALT) is slightly elevated (3–6 × upper limit of normal (ULN)), aspartate aminotransferase (AST) is normal, and 2D shear wave elastography (2D-SwE) showed no signs of fibrosis [2D SwE: 4.5 kPa, interquartile range (IQR) 1.5].

Patients in family B use special CN lamps by Dutch Medical Technology—kid's bed with LED lamps (maximal intensity 180 microWatt/cm^2^/nm) and portable LED lamps (adjustable intensity from 100 to 160 microWatt/cm^2^/nm). In the hospital setting, we confirmed that 10 h of PT a day, if consistently applied, is sufficient to keep the TB serum below neurotoxic levels. Unfortunately, in the last 6 months, their regulation of bilirubin was inadequate due to lower compliance with PT and frequent intercurrent febrile illnesses.

The pediatric neurologist and psychologist regularly assess all patients. Unfortunately, patient 1 developed kernicterus before LT. He is now 31 years old, has severe psychomotor retardation, and is entirely dependent on help from others. Other patients have normal psychomotor development and no neurologic impairment.

Regarding potential complications, none of our patients were diagnosed with cholelithiasis, cholecystolithiasis, or liver fibrosis. No one in our cohort developed light-induced erythema or skin cancer.

## 3. Discussion

The mainstay of treatment in our CNS patients was daily PT, as in previously reported studies (4, 8, 10, 11). Patients' TB levels were slowly but steadily increasing, which is unsurprising since it is well documented that PT becomes less effective with advancing age due to increased skin thickness, unfavorable body surface/weight ratio, and a decrease in compliance (10, 12). In our experience, which is in line with others (8, 10), PT represented the greatest burden to patients and their families, which invariably led to poor compliance. PT equipment has significantly improved over the years and LED lamps available today provide more effective PT with fewer side effects like heating. Nowadays, in a subset of highly motivated patients, PT alone can be sufficient even until adulthood (8), but the risk of irreversible neurologic sequelae persists. With potential new therapies like gene therapy on the horizon (4, 10), our primary goal is to optimize PT and postpone LT if possible.

LT is curative and indicated when TB levels rise to potentially neurotoxic levels despite optimal PT, and it should be performed before the onset of irreversible neurological sequelae (10, 13, 14). In the biggest reported cohort so far, 12.4% of CNS1 patients underwent LT at a median age of 9 years, which resulted in a marked reduction of TB (8). LT was performed in half of our patients (4/8) at the ages of 5.5, 7, 9, and 10. Two types of LT are performed in patients with CNS: orthotopic liver transplantation (OLT), where the entire liver is replaced with a whole or partial liver graft from a donor, and APOLT in which only part of the native liver is removed and replaced with a donated liver segment (10), with similar graft and patient survival rates reported in both transplant settings (15). Two of our patients underwent OLT; one died due to primary graft dysfunction, while the other has impeccable liver function 21 years later. Successful APOLT was performed in 2 patients who now have normal liver tests 13 and 11 years later. Immunosuppression protocols were the same in the patients who underwent OLT and APOLT.

A theoretical alternative to LT is hepatocyte transplantation (HT), in which 5% to 10% of the host liver is replaced with transplanted hepatocytes that provide the missing hepatic function once engrafted into the recipient's liver (16). In some CNS patients, HT increased UGT1A1 activity and resulted in up to 50% bilirubin reduction. Unfortunately, this effect was transient, and most of these patients underwent LT within a year (3, 16), which was also the case in our patient who received it.

The liver in CNS patients was traditionally considered structurally intact, which was recently refuted (17). Mildly elevated liver enzyme tests frequently found in CNS patients did not correlate with TB or UCB levels. However, higher AST was associated with advanced fibrosis in one-third of PT-dependent CNS1 patients older than 7 years (8). Our oldest patient currently treated with PT (6.5 years old, normal AST) has no signs of fibrosis according to two-dimensional sheer-wave elastography (2D-SwE US). Liver biopsy performed in patient 1 before LT (at the age of 9 years) showed no signs of fibrosis.

Our most exciting finding is the unusually high incidence of CNS in Croatia. With an incidence of 6.1 in 1 000 000 live births, Croatia is among the countries with the highest reported incidence of CNS. Our center is the only pediatric liver transplant center in Croatia; therefore, all CNS1 patients are referred to our center at some point. On the other hand, there may be patients with less severe CNS2 type that are not treated in our center and, therefore, not included in this study. Thus, we might have underestimated the incidence of CNS in Croatia.

So far, the highest incidence of CNS has been reported in Tunisia (8 in 1 000 000 newborns). They have an extremely high consanguinity rate (25%–60%) (8) and confirmed founder effect (18). CNS is similarly common in other communities with a high frequency of pathogenic UGT1A1 gene variants due to a founder effect, such as in Old Order Amish and Mennonite populations (4).

In Croatia, consanguineous marriages are uncommon, and our patients were from 5 families with no reported consanguinity. Nevertheless, there are some small isolated populations, for instance, on islands, and in distant villages, with reproductive isolation and specific genetic structure of their populations, characterized by a small gene pool, consanguinity, and inbreeding. As a result, extremely rare mutations and diseases they are causing are found in unusually high frequencies (19).

Four patients (including family B) are the offspring of two families originating in a small Croatian enclave in Kosovo called Janjevo, which can be recognized as one of the populations above. Janjevci are one of the oldest ethnic groups of Croats. They lived in Kosovo for seven centuries as a relatively isolated ethnic group, and then in the second half of the 20th century, most of them emigrated to Croatia. Other than relative isolation resulting in a small gene pool, another specific feature of this population is the high birth rate (20). Parents in family B refused prenatal testing, and all three of their children have CNS1.

Searching for a potential explanation for the higher-than-expected incidence, we analyzed *UGT1A1* mutations in our patients. To date, more than 130 *UGT1A1* mutations have been identified, and close to 100 of these were reported in CNS patients (21). CN1 results from genetic changes that cause premature truncation or critical amino acid residue substitution (21).

Patients 3 and 4 (family A) are homozygous for a frameshift mutation, c.717_718delAG; p.Q239fsX256 that introduced a premature stop codon at position 256. Genetic analysis was performed in Italy, and our patients were included in the cohort of 31 patients (15 CNS1 and 16 CNS2 patients), where the same mutation in heterozygous form was also found in 2 CNS2 patients of Italian origin (22). It was also found in CN1 patients in Slovakia (23).

Patient 5 is homozygous for a frameshift deletion mutation c.722_723delAG; p.Glu241GlyfsTer16 in exon 1, which leads to the expression of a truncated protein, and was previously reported in an Italian patient (24).

Patients 6, 7, and 8 (family B) are homozygous for the variant designated UGT1A1*10 (25) c.1021C >T; p.(Arg341*) in exon 3, which results in the replacement of arginine with a stop codon at position 341. This variant has previously been reported in Pakistani (26) and Chinese patients (27) born to consanguineous parents.

In 6 patients from 3 families with known mutations, we found 3 different mutations that were not previously described in any population similar to ours. Nevertheless, we can assume that our population has no significant founder effect.

Undeniably, higher than expected incidence in Croatia can, to some extent, be attributed to the fact that our small cohort includes 5 patients from only 2 families. Therefore, the allele frequency may not be that much higher than in other populations. Nevertheless, this alone cannot explain the incidence 6 times higher than expected, especially since, in cases of inherited conditions like CNS, the familial clustering is inevitable and present in other cohorts as well.

There are considerable downsides to our study. Medical documentation of some patients was incomplete.

## 4. Conclusion

This is the first comprehensive study investigating a cohort of Croatian patients with CNS1. Our experiences do not significantly differ from others, with long-term PT and LT as the mainstays of treatment. We tried to find an explanation for an unusually high incidence of CNS1 in Croatia and analyzed UGT1A1 variants found in our cohort. The only credible clue is that half of our patients are descendants of an isolated enclave in Kosovo with a small gene pool and a high potential for inbreeding.

## Data Availability

The original contributions presented in the study are included in the article/Supplementary Material, further inquiries can be directed to the corresponding author.
